# Development of clinically relevant orthotopic xenograft mouse model of metastatic lung cancer and glioblastoma through surgical tumor tissues injection with trocar

**DOI:** 10.1186/1756-9966-29-84

**Published:** 2010-06-29

**Authors:** Xi Feng Fei, Quan Bin Zhang, Jun Dong, Yi Diao, Zhi Min Wang, Ru Jun Li, Zi Cheng Wu, Ai Dong Wang, Qing Lan, Shi Ming Zhang, Qiang Huang

**Affiliations:** 1 Neurosurgical Department, Brain and Nerve Research Laboratory, The First Affiliated Hospital of Soochow University, 188 Shizi Street, 215006, Suzhou, China; 2 Neurosurgical Department, Brain Tumor Research Laboratory, The Second Affiliated Hospital of Soochow University, 1055 Sanxiang Road, 215004 Suzhou, China; 3 Department of Pharmacology and Laboratory of Aging and Nervous Diseases, Soochow University School of Medicine, 199 Renai Road, 215123, Suzhou, China; 4 Neurosurgical department, Suzhou Kowloon Hospital of Shanghai Jiaotong University School of Medicine, 118 Wansheng Street, 215021, Suzhou, China

## Abstract

**Objective:**

Orthotopic models are important in cancer research. Here we developed orthotopic xenograft mouse model of metastatic lung cancer and glioblastoma with a specially designed system.

**Methods:**

Tiny fragments of surgical tumors were implanted into the mice brain with a trocar system. Immunohistochemistry was performed to detect brain tumor stem cells among glioblastoma tissues, including both the original and resulting ones with monoclonal antibody against CD133.

**Results:**

Besides the constant high take rates in both models; brain transplants perfectly resembled their original tumors in biological behaviors. The brain tumor stem cells, positively stained with CD133 were found, though not frequently, in both original and resulting glioblastoma tissues.

**Conclusions:**

Orthotopic model established with a trocar system is effective and injection of tumor tissues containing stem cells promise the forming of new tumor mass when grafted.

## Background

Animal models have been extremely critical in the understanding of cancer and in the pre-clinical testing of new antitumor drugs since 1960s when it was first developed by implanting human colon carcinoma to nude mice [1]. The utility of each particular model, nevertheless, depends on how close it replicates the original tumor. To the present days, several kinds of animals, like dog, monkey, and murine, have ever been tested and compared between each other for the purpose of finding the best host for transplantation [2-4]. The results indicated that though the extent to which murine models recapitulate the features encountered in human tumor is still controversial, considering their reproducibility and availability, they still constitute a valuable in vivo system for the preclinical studies.

Not surprisingly, an orthotopic model is much more superior to a heterotransplantation model in that the former recapitulates the original tumor more likely. As far as human brain tumors are concerned, the orthotopic models currently available are established either by stereotaxic injection of cell suspensions [5-8] or implantation in solid piece through complicated craniotomy [9,10]. Taking into consideration both the advantages and disadvantages of the current methods, there is still much room for improvement. Recently, high success rate of model development of brain tumor were established using cell suspensions directly derived from fresh patient brain tumors indicating the important role of stromal cells in tumor formation [11].

In the current study, we developed orthotopic xenograft mouse model by injecting tiny tumor tissue, but not cell suspensions, into the brain of mouse with a special trocar system. It is argued that the organ-specific microenvironment plays a determining role in the growth patterns of transplanted tumors [12,13]. To observe the growth patterns of different tumor types implanted to the same organs, we chose primary glioblastoma multiforme and brain metastasis for transplantation in this study. The growth of xenografts in the mice brain was observed with MRI. Histological study was also performed to explore and compare the growth features of these two biologically distinctive malignances. With the identification of CD133 positive cells from brain tumor tissues, more and more reliable evidences support the assumption that CD133^+ ^cell is the tumor initiating cells or cancer stem cells[14-16]. In this study, we also examined the distribution of CD133^+ ^cells in both the original and implanted tumors of glioblastoma multiforme.

## Methods

### Brain tumor specimens

Our study was approved by the Medical Review Board of Soochow University Medical School. The donor tissues obtained at surgery after written consent consisted of typical glioblastoma multiforme (WHO classification 2000) and brain metastasis from lung adenocarcinoma. Tumor tissue was dissected free of blood clot, washed, and minced into 0.5-mm-thick slices for grafting.

### Reagents and equipments

Alcian blue/PAS dyeing reagent was provided by pathology department of our hospital; Rabbit anti-carcinoembryonic antigen (CEA) monoclonal antibody, horseradish peroxidase(HRP), and 3,3'-Diaminobenzidine(DAB) were offered by pathology department of Changhai hospital, affiliated hospital of the second military medical university; EGFR((BDbioscience Co.); CD133((Miltenyi Biotec); 24# trochar(B. Braun Melsungen AG); Micro-drill 18000-17(Fine Science Tools); Supraconduction nuclear magnetic resonance formatter equipped with micro-23 windings(Philips Achieva).

### Animals

Four to six-week-old male and female NC nude mice at an average weight of 25 g were purchased from the Center for Experimental Animals, Soochow University (certificate No. SY X K (Su) 2007-0035). All the animals were bred and maintained in the Specific Pathogen Free Animal Care Facility, Nasal1000 grade. The National Institutes of Health guidelines for the care and use of laboratory animals were followed in all animal procedures.

### Orthtotopic tumor tissue transplantation and further propagation

**Figure 1:**
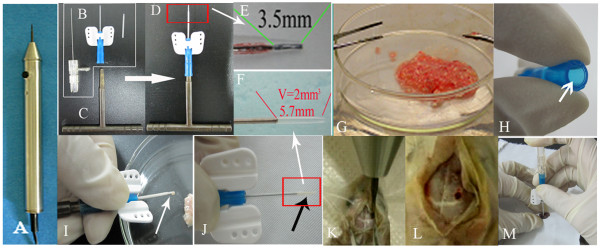
**Illustration of nude mice orthotopic transplantation with glioma tissue**. A: micro-skull drill; B: trochar; C tissue propeller; inset in D and E: the depth of injection into mouse brain; G comminuted tumor tissue; H put some tissue into the rear part of trochar (see arrow); I: tumor tissues was packed to the trochar cannula with propeller for transplantation, superfluous tumor tissue were overflowed from the distal end of trochar under the pressure of propeller (see arrow);F and inset in J: exactly 2 mm^3 ^tumor tissue lefted for transplantation (see black arrow); K: drill the hole; L:the burr hole; M: the tumor tissue (J) was injected slowly into brain via the hole (I), then pulled out the trochar slowly, sealed the hole with bone wax and sutured the scalp.

For transplantation, we designed a very simple but ingenuous injection system. This system includes a 24# trocar and a specifically made propeller. The propeller matches well with the rear part of the trocar and is used to pack the tumor tissue in the trocar cannula. When the trocar filled with tumor tissue is navigated by stereotaxic instrument to the injection destination, trocar needle was introduced to slowly and smoothly push tumor tissue out. The injected volume could be strictly controlled according to the length on the cannula which is quantitated by 2 mm^3 ^water (Figure 1). In this study, all the surgical procedures were carried out under general anesthesia by intraperitoneal injection of 10% chloral hydrate (200 mg/kg). A small burr hole, 2 mm in diameter was made 2 mm to the midline and 0.5 mm anterior to bregma using micro-skull drill. Trochar packed with donor tissue was navigated to a depth of 3.5 mm via skull hole. Tumor tissue, 2 mm^3 ^per mouse, was slowly and smoothly injected into the caudate/putamen nuclei of the mouse brain. Skull hole was sealed with bone wax and scalp sutured. The implanted tumors in the mouse brain were passed from animal to animal following the same procedure described above for six generations in the metastasis group (15 mice for the first generation and 10 mice for the other generations) and thirteen generations in glioblastoma multiforme group (10 mice per generation). Take rate of each model and survival time of each mice were counted. As mice usually died in 2 days after cachexia occurs, survival time of tumor-bearing mice was calculated as 1 day + days from transplantation to sacrifice.

### Magnetic resonance imaging (MRI) of nude mice implanted with tumor tissues

After anesthetized as the same way described above, mice were fixed in micro-23 winding mice MRI equipment. A 1.5 T clinical Signa version 5.5.1. (General Electric MS) was used for brain imaging. Five apparently normal mice were examined on day 10, 15, 20, 25 and day 30 post tumor implantation to detect the growth of the grafted tumor fragments. In enhanced scanning, 0.5 ml diethylene triaminepentaacetic acid gadolinium (Gd-DTPA 0.25 mmol/L) was intraperitoneally injected 10 minutes before examination. Scanning parameters was as follows: MATRIX 224X224; layer thickness: 3.0 mm; space between layers: 0.3 mm T1WI: TR260ms and TE24ms.

### Histological examination

Four mice that received orthotopic implantation of human glioblastoma multiforme were sacrificed on day 5, 10, 15, or 20 to study brain tumor take. The other mice were sacrificed when they became cachectic or at various post-implantation times for morphological studies. The overview of tumor mass and its relationship with adjacent host brain structures was observed with a naked eyes or low power lens. The brain tissues harboring xenografts were fixed in 4% phosphate-buffered paraformaldehyde for 18 hours, embedded in paraffin. Sections of all paraffin-embedded blocks were stained with hematoxylin-eosin (HE) and with Alcian blue/PAS. As CEA is the potent marker for lung adenocarcinoma and EGFR is specially expressed in glioblastoma multiforme, we also performed immunohistochemistrical staining to examine the expression of CEA and EGFR in xenografts derived from metastatic adenocarcinoma or glioblastoma multiforme.

### The CD133 expression in the original human glioblastoma and its transplants

CD133^+ ^tumor cells are rare among tumor tissues, but regarded as the initiating cells in the brain tumor formation. In this study, the glioblastoma tissues used for and resulted from orthotopic implantation were obtained processed and immunostained for CD133 expression as described by Christensen et al [17].

## Results

### Efficient transplantation and high take rates were achieved

Due to the improvement of procedure, it took only about 5 minutes to finish the implantation (from anesthesia to closure of skull hole) in one mouse. Moreover, no postoperative death happened. None of the mice with xenograft developed focal neurological signs in the early and intermediate periods, however, at the end of observation, all the tumor-bearing mice presented with reduced food intake, dull response, emaciated figure, skin fold and cachexia. The take rates in brain metastasis group increased gradually, with 33% for first generation, 50% for the second generation, 70% for the third generation, and 100% from the 4th generation (table 1). In glioblastoma group, the results were even more encouraging with success rates of 90% for the first and second generations. From 3^rd ^generation, the tumorigenicity rate was steadily up to 100% (table 2). Survival time of mice with metastasis grafts varied considerably from mouse to mouse of the first three generations, but tended to be similar from the 4th generation (38.0 ± 0.9 days n = 10, see table 1). Mice in the glioblastoma group demonstrated the same tendency, having a survival time of 23.9 ± 1.7 days (see table 2) from the 5th generation (n = 10).

**Table 1:** Take rates in brain metastasis group and survival time of tumor-bearing mice.

Generation	No. of mice	No. of tumor-bearing mice^1^	Take rate(%)	survival time(d)
1	15	5	33	47.6 ± 1.8
2	10	5	50	42.2 ± 1.8
3	10	7	70	40.8 ± 1.2
4	10	10	100	38.0 ± 0.9
5	10	10	100	38.6 ± 1.0
6	10	10	100	37.8 ± 0.9

^1^Each mouse was implanted with one graft (Site: right caudate nucleus of nude mice)

Take rates in brain metastasis group from lung adenocarcinoma and survival time of tumor-bearing mice in the intracranial xenotransplantation

**Table 2:** Take rates in glioblastoma group and survival time of tumor-bearing mice.

Generation	No. of mice	No. of tumor-bearing mice^1^	Take rate(%)	survival time(d)
1	10	9	90	32.4 ± 2.1
2	10	9	90	30.4 ± 2.2
3	10	10	100	29.9 ± 2.1
4	10	10	100	28.4 ± 2.7
5	10	10	100	23.9 ± 1.7
6	10	10	100	23.0 ± 0.9
7	10	10	100	22.8 ± 1.3
8	10	10	100	21.7 ± 1.3
9	10	10	100	23.2 ± 0.6
10	10	10	100	22.0 ± 1.8
11	10	10	100	21.3 ± 1.2
12	10	10	100	21.4 ± 1.8
13	10	10	100	22.4 ± 0.9

^1^Each mouse was implanted with one graft(Site: right caudate nucleus of nude mice)

Take rates in glioblastoma group and survival time of tumor-bearing mice in the intracranial xenotransplantation

### Implanted tumors could be revealed by MRI

**Figure 2:**
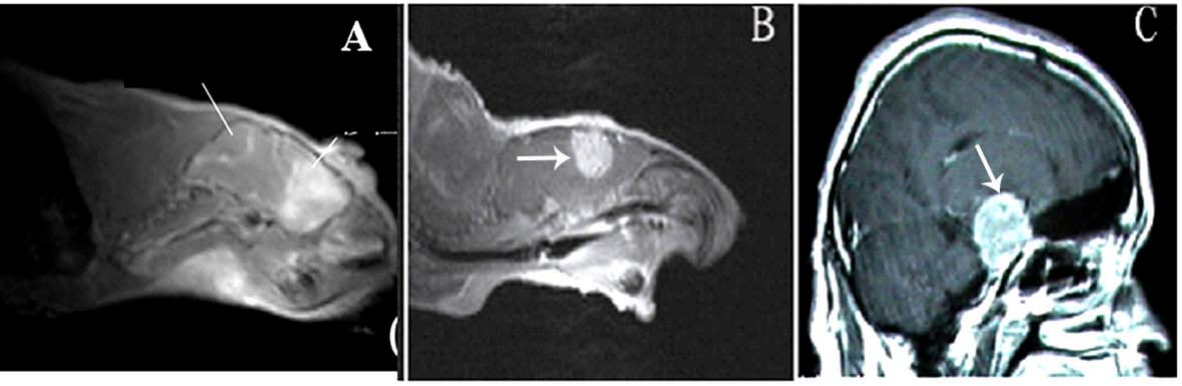
**Orthotopic xenografts in brain of mice revealed by MRI**. A + B: the border of the orthotopic graft of human glioblastoma (white lines) was vague (A), in contrast to the sharp and clear edge of orthotopic graft of human brain metastasis (B white arrow). Post-Gd-DTPA sagittal T1W sequences revealed a typical enhancement in both A and B; C:Post-Gd-DTPA sagittal T1w sequences image of clinical case with brain metastasis of human lung adenocarcinoma(white arrow). The image was very similar to B.

MRI scanning revealed tumor mass as early as day 20 for metastasis group, and day 15 for glioblastioma multiforme. The imaging features of xenograts from brain metastasis were apparently different from those of xenografts from gliomblastoma multiforme. The former has a distinct boundary with adjacent normal parenchyma, while glioblastoma multiforme was featured by vague border and finger-like edema (Figure 2). Post-Gd-DTPA sagittal T1W sequences revealed a typical enhancement in both malignances.

### Gross morphology

**Figure 3:**
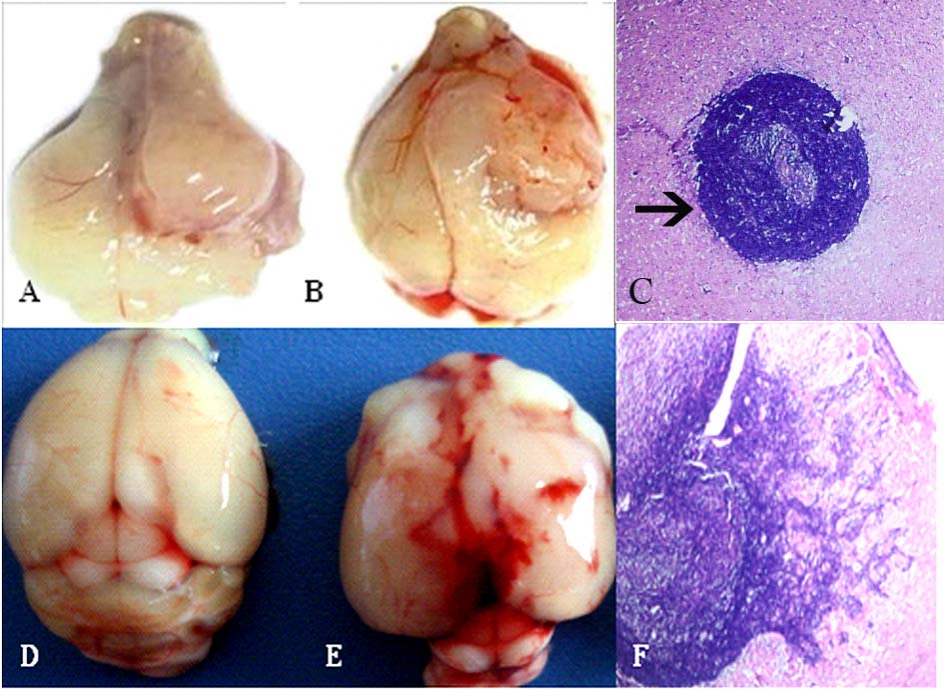
**Brain of tumor-bearing mice observed by eyes and under lower power lens**. A-C: brain metastasis tissues was implanted in right caudate nucleus. Tumor had grown to the brain surface of right hemisphere. The boundary between tumor and normal tissues was very clear seen by eyes (A and B) or under microscope(C arrow). D-F: the transplantation position of glioma was right caudate nucleus too. There was no tumor can be seen on the surface but brain edema was apparent. Under microscope Tumor cells were seen extensively invading to adjacent brain tissues.

Xenografts derived from brain metastasis were gray, soft and featured by sharp boundary with adjacent normal parenchyma. In glioblastoma models, tumors were gray or yellowish, measuring from 6 to 8 mm in largest diameter. Besides invasion to ipsilateral hemisphere, contralateral spread was also observed though it was not frequent. Extension of tumor mass to the skull and scalp soft tissue was not found (Figure 3).

### Histopathologic examination of implanted tumors

**Figure 4:**
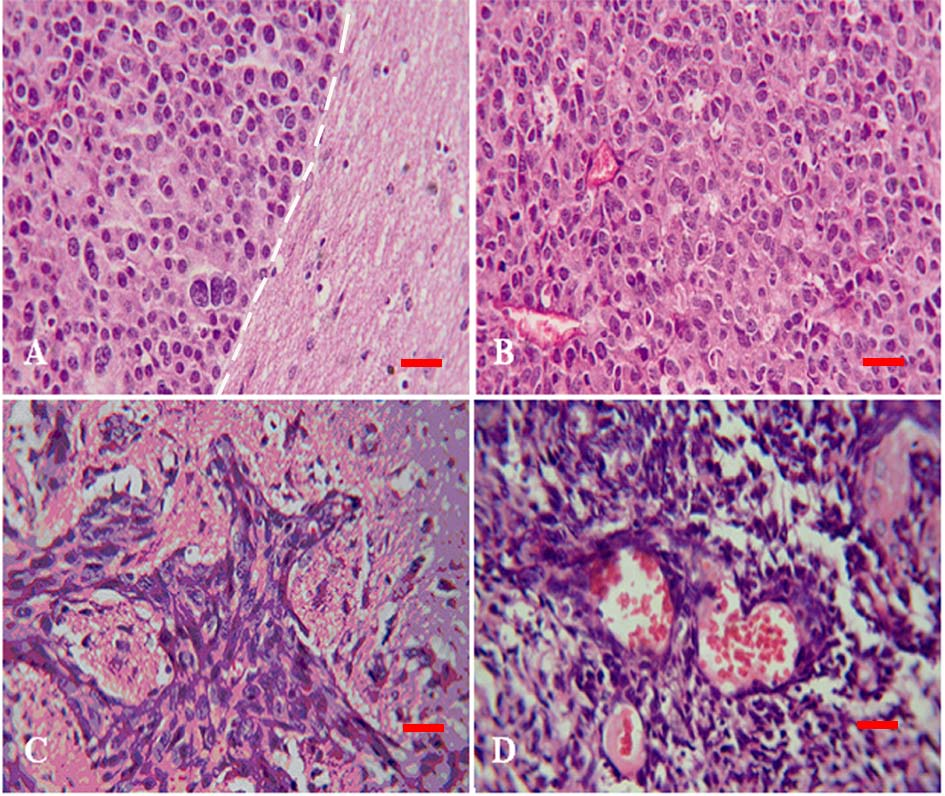
**Transplantation tumor observed by HE staining**. Tumor cells of brain metastasis (A and B) were small round, easy to see caryocinesia, rare to see multinucleated giant cell and did not form glandular cavity in somewhere (B). Boundary (white dash line) between tumor (left) and normal brain tissues (right) was very clear (A). There was no apparent boundary can be seen between glioma tumor and surrounding brain tissues (C and D) and tumor cells invaded like chicken wire. Tumor cells were fusifirm, star-like, triangle and so on. Abundant vessels shown in tumor tissues and the dndothelial cells were hyperplasy (D).

**Figure 5:**
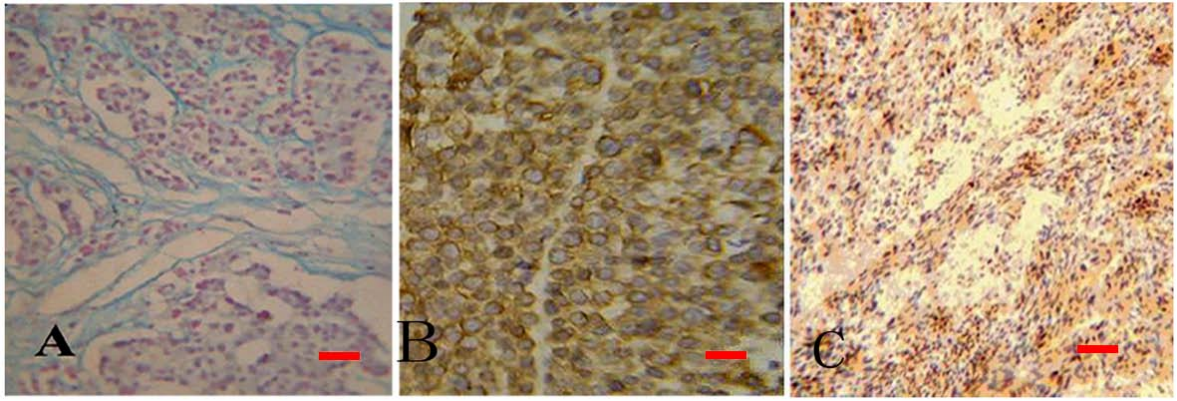
**Markers expressed in xenografts of brain metastasis**. A: stroma was stained deep blue with Alcian blue staining indicating mucus secreted by tumor cells was acid. B: immunochemistry of CEA in brain metastasis showed nearly all tumor cells highly expressed CEA compared to normal tissues. C: immunochemistry of EGFR in glioma indicated most tumor cells expressed EGFR.

In HE sections, features common to xenografts of brain metastasis included: a) sharp boundary between tumor mass and surrounding normal brain tissue (Figure 4A and 4B); b) round and densely arranged tumor cells; c) abundant caryocinesia; d) abundant acid mucus secretion by tumor cells that were dyed blue by Alcian blue and red by PAS; e) positive immunostaining for CEA (Figure 5A and 5B). Obviously, the transplantation of brain metastasis tissues into the nude mice brain produced tumor mass which perfectly recapitulated the original tumor type. In contrast to the xenografts derived from brain metastasis, the resulting tumors from human gliomblastomas demonstrated variable cytoplasmic and nuclear pleomorphism on the preparations. Cellular forms ranged from fusifirm, starlike to triangle with scant cytoplasm and densely hyperchromatic nuclei. Bizarre, multinucleated giant cells were frequently observed. Exuberant endothelial proliferation in combination with necrosis was significant (Figure 4C and 4D). EGFR, one of the important markers for glioblastioma multiforme, was strongly expressed on membrane and in cytoplasm of tumor cells (Figure 5C).

### CD133 + cells were seen in both the original tumors and the implanted tumors

**Figure 6:**
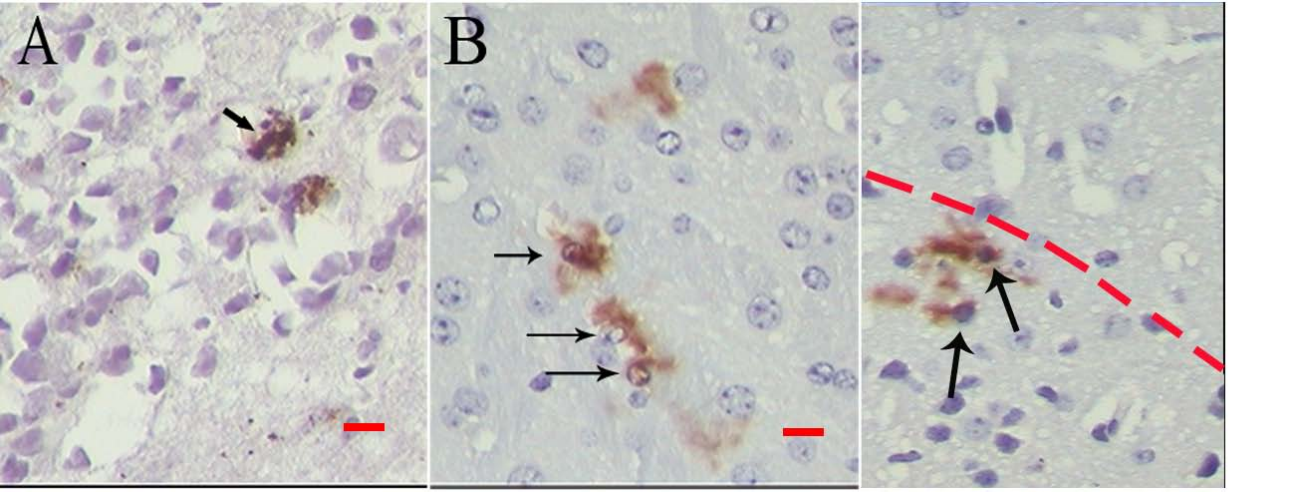
**CD133 expressed in both original tumors and the implanted tumors**. Tumor sections were stained against human specific CD133 by common immunochemistry, rare cells were positive for CD133 both in original tumors (A) and transplantation tumors (B). It could also be seen that CD133 positive cells distributed at the border (red dash line) between tumor mass(bottom) and the adjacent brain parenchyma (top in C).

Immunohistochemical staining for CD133 protein was performed in sections made from the original glioblastoma multiforme and its successive xenografts. As a result, CD133 positive cells were rare but observed in each tumor tissue. It is rather intriguing that CD133 positive cells were prone to distribute at the border between main tumor mass and the adjacent normal brain parenchyma (Figure 6).

## Discussion

In the previous published orthotopic animal models of brain malignances, the tumors were transplanted by cell suspension injection [5-8] or surgical implantation via craniotomy [9,10]. Cell suspension injection has once been widely adopted due to the distinctive advantage of micro-invasion. However, to acquire single cell suspensions, trypsin is usually added to dissociated tumor tissues or adherent cell lines, which inevitably reduced the viability of the tumor cells. Secondly, because of the small cranial cavity of mouse, the total volume of injected cell suspension is limited to or less than 20 μl [5-8], which means the relatively small number of could-be implanted tumor cells. Furthermore, cell suspensions are deprived of stromal component which is actually critical in the tumor growth. Based on these listed reasons, it is not surprising that implantation of tumor cell suspension resulted in an overall take rate of less than 70% despite the recent refinery in transplantation procedure. Partly because the injection pressure or speed couldn't be well controlled, the tumor cell suspension may flow into the ventricle, and/or flow back along the shaft of the needle into the arachnoidal space. This may account for why clinically GBM metastasis rarely happen, but most human GBM tumor cell lines intrinsically possess metastatic potential. Moreover, GBM models produced by most cell lines without stromal component always failed to invade the contiguous brain, growing by rather expansive than diffusely infiltrative pattern. Taken together, from the take rate to the recapitulation potentials, animal model via cell suspension injection of established cell lines seems far from desirable.

Tumor implantation in solid piece is theoretically superior to cell suspension injection in the following aspects: 1) when the transplantation volume is same, solid piece contains tumor cells almost 20 times more than cell suspension does; 2) besides the tumor cells, the stroma was implanted at the same time, which provides a microecosystem that favorites the cell growth and the maintenance of the biological features of original tumors. Tumor transplantation in solid piece was firstly reported by Shapiro et al [18], however, the success rate is unexpectedly low, with an overall take rate of 16% for human grade II-IV astrocytomas, and 24% for GBMs. Recently, Antunes et al [10] significantly improved the take rate by indirect transplantation of human glioblastoma; however, he also observed extracranial extension and scalp soft tissue infiltration of the resulting tumors, which never happens clinically. Considering the trauma to the mice, the complicated procedures, and other problems, tumor fragment grafting via craniotomy still has much room for improvement.

Enlightened by the advantages of cell suspension injection and disadvantages of tumor fragment grafting, we designed to implant tumor in solid piece through injection. It is a simple but ingenious modification which resulted in the following advantages in our model when compared with implantation via craniotomy: 1) being minimally invasive as only a very small skull hole is enough; 2) high efficiency due to the simplified manipulation; 3) being highly homogeneous, especially in survival time as the volume of implantation could be strictly controlled; 4) no extracranial extension of tumor mass, which is sometimes though not frequently encountered in cases of craniotomy; 5)more reasonable mean survival times of 38 days for metastasis model and 24 days for glioblastoma mutiforme model. In some GBM mouse models via craniotomy [10], the mean survival time is as long as one year, which is absolutely beyond the rational ranges when the survival time of a patients with brain metastasis or glioblastoma multiforme and the average expectation life time of a tumor-spared mouse are taken into consideration.

Operative mortality in preliminary experiment was high to 16.7%, some died because of traumatic intracranial hemorrhage during operation, and other died because of encephaledema after operation. We do our best to reduce the surgical trauma to a minimum and make zero death rates in perioperative period, though there was some operation-related mortality in the preliminary experiments. These deaths were mainly due to traumatic intracranial hemorrhage and/or brain edema after operation. To avoid these accidents, we took the following measures: 1) 22# trochar was replaced by 24# trochar; 2) transplantation volume was reduced to 2 mm^3^; and 3) the tumor tissues were pushed as smoothly as possible.

Take rate is not the only criterion in evaluation of an orthotopic animal model, while how close a model can replicate the original tumors is more essential. As brain metastasis and primary glioblastioma are two biologically different malignances in the central nervous system, we selected them both as grafts in this study to assess this novel method. When compared between the two models, metastasis xenografts were evidently differentiated from glioblastoma xenograft in many aspects, however, when compared with their original malignances, both models demonstrated unquestionable similarity in histological structure features and growth patterns. Laurent et al. [10] performed both heterotransplantation and orthotopic transplantation of human glioblastoma, and concluded that the organ-specific environment play a determining role in growth and invasive properties. In the current study, two different malignances were transplanted into the same organ; however, the resulting tumors didn't demonstrate the similar growth patterns. So, it is more plausible and acceptable that it is the malignance itself but not environment that plays a determining role in the tumor growth patterns and other biological behaviors.

With the identification of brain tumor stem cells from tumor mass or cell lines, it is reported that as rare as 10^2 ^CD133+ glioma cells could generate tumor mass, while as much as 10^6 ^CD133- glioma cells failed to form tumor mass after injected to the mouse brain. The fact that cell suspension injection of most established cell lines often yields well-circumscribed intracranial tumors which are different from the original tumor, coupled with the complicated procedure of cell suspension injection precludes tumor stem cells as a desirable transplant [19-21]. In this study, the immunohistochemistry with monoclone antibody against CD133 revealed that not only the original tumors, but the resulting tumors were positively stained for CD133. This result means the tumor tissues contained brain tumor stem cells and functioned as a tumor stem cell pool. It is reported that biological behaviors of tumor stem cells are highly dependent on their microenvironment [22,23], in another word, CD133 negative tumor cells and stromal components also play an important role in the potential of tumor stem cells to re-establish the original tumor. Taken together, tumor stem cells, other tumor cells and stromal components make a concerted contribution to the growth of tumor mass in transplantation animal model.

## Conclusions

In conclusion, orthotopic xenograft mouse model of metastatic lung cancer and glioblastoma was established successfully by our specially designed trocar implantation system and it makes the orthotopic transplantation of brain tumor into nude mice simpler, easier but more efficient. When a large amount of homogeneous animal modes are required in experiments, especially in new antitumor drug tests, this method of tumor tissue injection promises the capacity to meet the demands.

## Competing interests

The authors declare that they have no competing interests.

## Authors' contributions

YD and RJL build the animal model. XFF, YD and ZCW carried out the immunoassays. ADW participated in the design of the study and performed the statistical analysis. QH, ZMW and QL conceived of the study, and participated in its design. XFE, QBZ, SMZ and JD helped to draft the manuscript. All authors read and approved the final manuscript.
